# Supplementary material for: Exosomes derived from highly scalable and regenerative human progenitor cells promote functional improvement in a rat model of ischemic stroke
Source: bioRxiv. 2025 Jan 10:2025.01.07.631793. Preprint. [Version 1] doi: 10.1101/2025.01.07.631793 (PMC11741374; doi:10.1101/2025.01.07.631793)
Supplement: Supplement 1 [file NIHPP2025.01.07.631793v1-supplement-1.pdf]

**Suppl Fig 1.** Comparison of functional activity in three eEPC exosome lines (eEPC1-exo, eEPC2-exo and eEPC3-exo) isolated from TFF-SEC vs. SEC only, (A) Scratch wound healing assay and (B) Live cell tube formation assay

**A. Scratch wound healing assay**

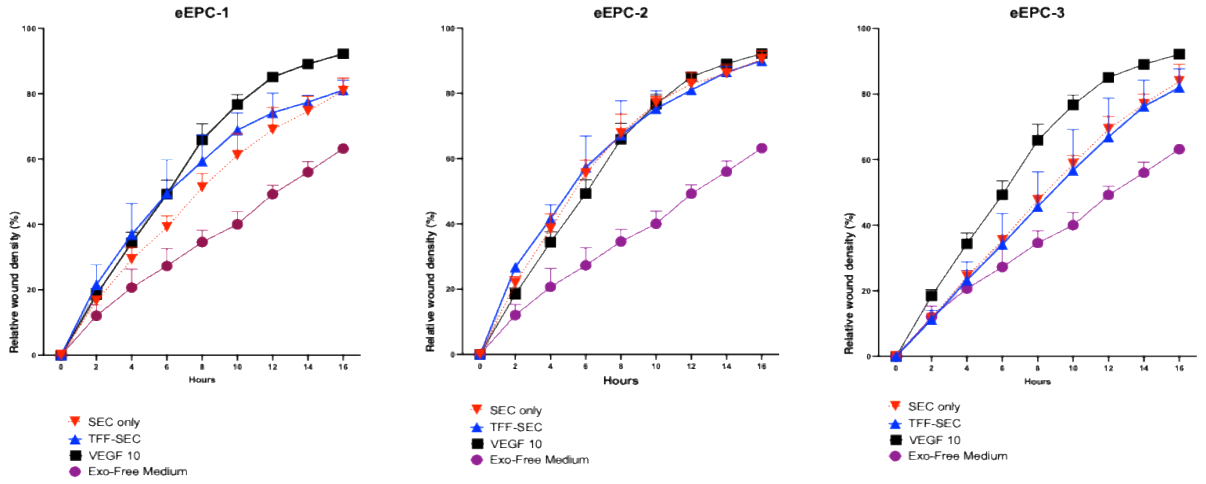


**B. Live cell tube formation assay**

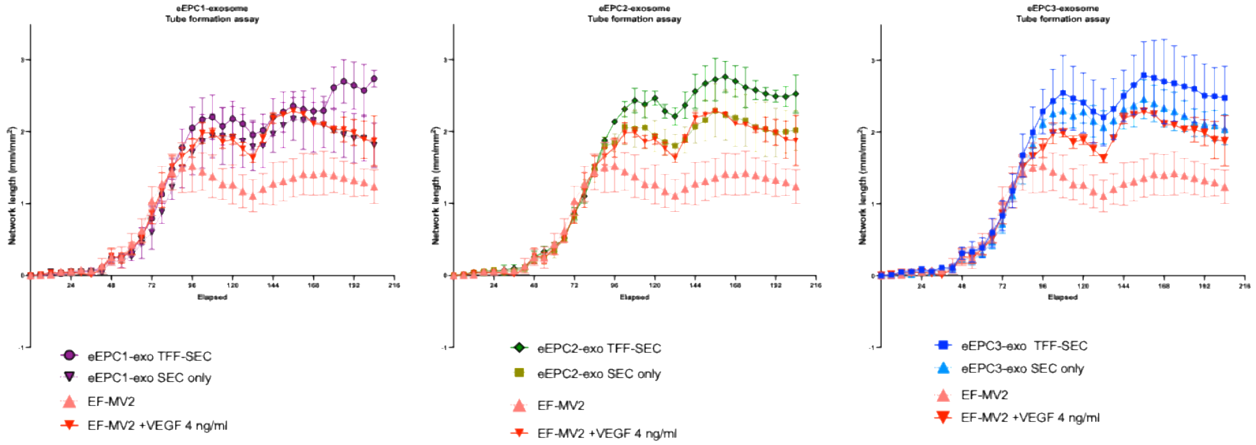

**Suppl Fig 2.** Migration assays for (A) Dose Response of eEPC-exosomes, (B) Comparison of bioactivity in eEPC-exosomes vs. Fibroblast-exosomes, and (C) Test of inactivated exosome.

**A. Dose Response of eEPC-exosomes**

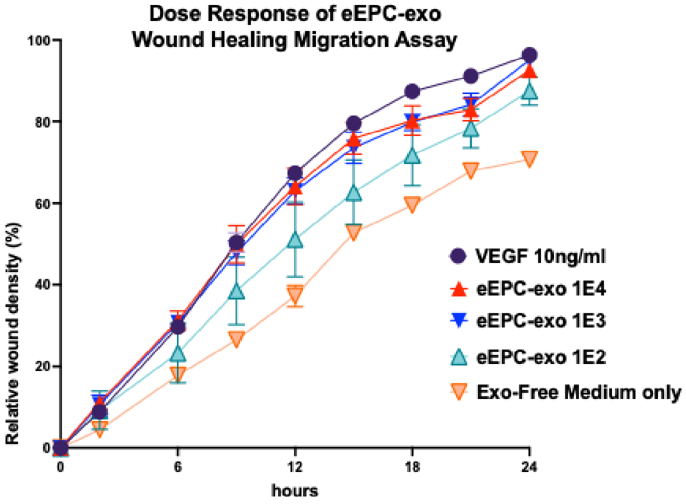


**B. Comparison of bioactivity in eEPC-exosomes vs. Fibroblast-exosomes**

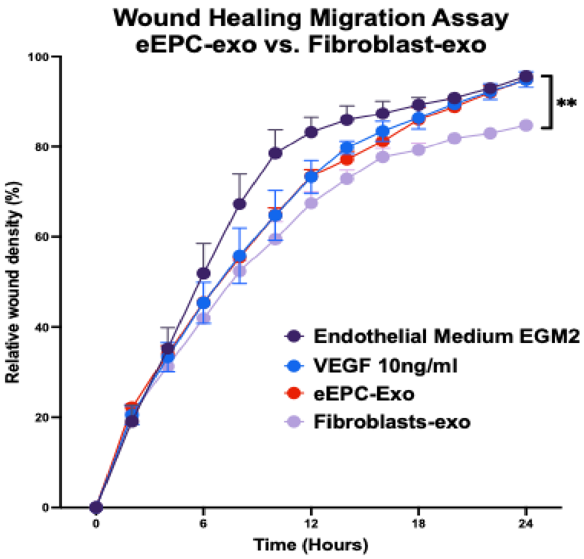


**C. Test of inactivated exosome**

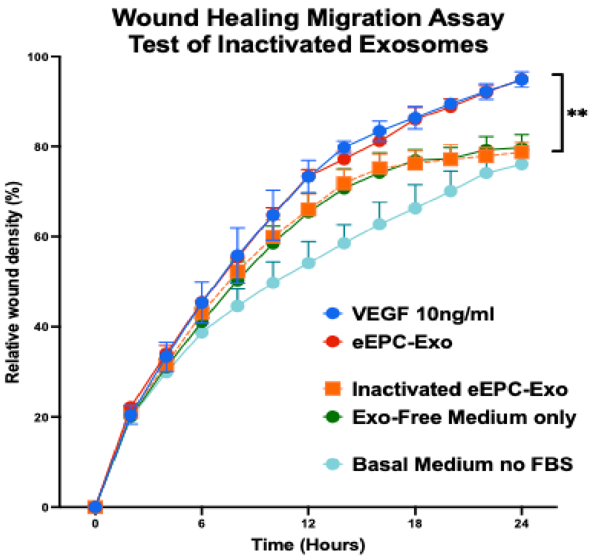

**Supple Fig 3.** Gene Ontology Analysis of eEPC2-Exosome Small RNA-seq.

**Gene Ontology (GO) Analysis pf targets of top 50 miRNAs (p>0.05)**

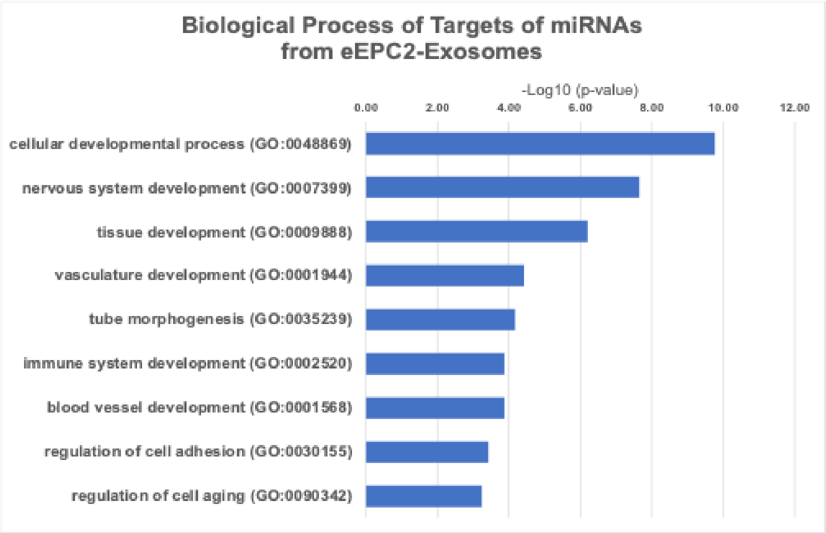

**Suppl Figure 4.** Mass spectrometry protein analysis of top 36 abundant protein in eEPC2-exosomes. (A) Interaction network of top 36 proteins in eEPC2-exosomes, (B) Gene ontology analysis of biological process in eEPC2-exo, and (C) List of proteins enrichment network.

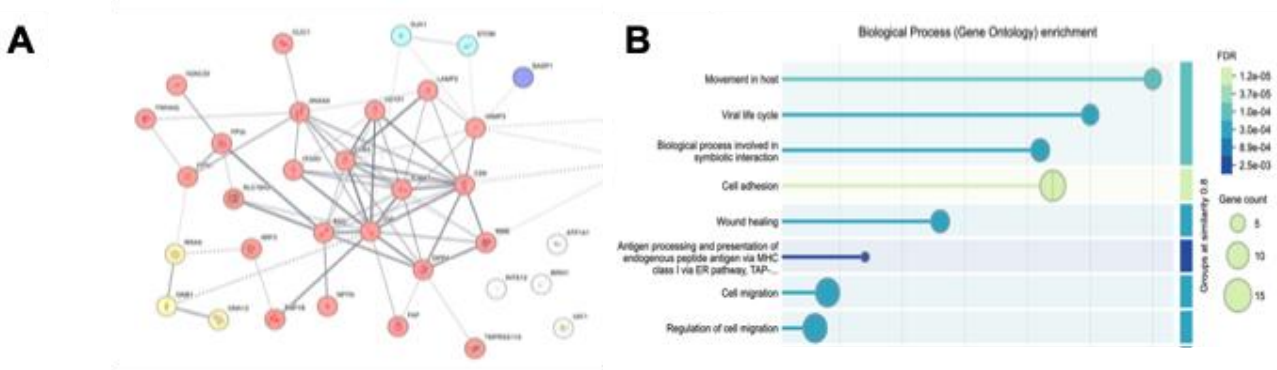


**C**

| GO term ID | term description | false discovery rate (FDR) | matching proteins in your network (labels) |
|---|---|---|---|
| **GO:0007155** | Cell adhesion | 0.00013 | VAMP3,FAP,ITGAV,ICAM1,NPTN,CD151,BSG,DPP4,CLIC1,CD9,ITGB1,PPIA,CD63 |
| **GO:0044000** | Movement in host | 0.0018 | ITGAV,ICAM1,BSG,DPP4,ITGB1,PPIA |
| **GO:0016477** | Cell migration | 0.0021 | FAP,RRAS,ITGAV,ICAM1,CD151,BSG,DPP4,CD9,ITGB1,PPIA,CD63 |
| **GO:0019058** | Viral life cycle | 0.0021 | ITGAV,ICAM1,BSG,DPP4,ITGB1,PPIA |
| **GO:0030334** | Regulation of cell migration | 0.0021 | PFN1,RRAS,ITGAV,ICAM1,GNA12,GJA1,CD151,BSG,DPP4,CD9,ITGB1 |
| **GO:0044403** | Biological process involved in symbiotic interaction | 0.0021 | ITGAV,ICAM1,STOM,BSG,DPP4,ITGB1,PPIA |
| **GO:0042060** | Wound healing | 0.0029 | GNA12,ANXA5,CD151,CLIC1,CD9,ITGB1,PPIA |

**Suppl Table 1**. List of most abundant miRNAs from small RNAseq analysis in eEPC-exo lines.

| | eEPC1-exo | eEPC2-exo | eEPC3-exo | miRNA target(s) | Function |
|---|---|---|---|---|---|
| hsa-miR-126 | ✓ | x | ✓ | Spred-1 | EC-mediated angiogenesis |
| hsa-miR-192 | ✓ | ✓ | ✓ | SIP1 | Remodeling |
| hsa-miR-92a | ✓ | ✓ | ✓ | ITGB5 | EC-mediated angiogenesis |
| hsa-miR-130 | ✓ | ✓ | ✓ | GAX, HOXA5 | EC-mediated angiogenesis |
| hsa-miR-132 | ✓ | ✓ | ✓ | p120RasGAP | EC-mediated angiogenesis |
| hsa-miR-221/222 | ✓ | ✓ | ✓ | c-kit, eNOS*, p27/Kip1 | EC-mediated angiogenesis |
| hsa-miR-155 | ✓ | ✓ | ✓ | SHIP1, SOCS1, IL12 | Wound Healing: Control of inflammation |
| hsa-miR-20a | ✓ | ✓ | ✓ | VEGF, E2F1 | Wound Healing: EC-mediated angiogenesis |
| hsa-miR-17-5p | ✓ | ✓ | ✓ | TIMP1 | EC-mediated angiogenesis |
| hsa-miR-20a | ✓ | ✓ | ✓ | E2F1 | EC-mediated and tumor induced angiogenesis |
| hsa-miR-378 | ✓ | ✓ | ✓ | Sufu, Fus-1 | Angiogenesis |
| hsa-miR-155 | ✓ | ✓ | ✓ | AT1R | EC-mediated angiogenesis |
| hsa-miR-210 | x | ✓ | x | ISCU1/2, E2F3 | Re-epithelization, EC-mediated and tumor induced angiogenesis |
| hsa-miR-29b/c | x | ✓ | x | Smads, beta-catenin | Wound Healing: Remodeling |
| hsa-miR-21-5p | ✓ | ✓ | ✓ | PTEN, PDCD4 | Control of inflammation and apoptosis |
| hsa-miR-146 | ✓ | ✓ | ✓ | IPAK, COX2 | Control of inflammation |

**Suppl Table 2.** Mass spectrometry protein analysis list of (A) top 27 abundant protein in eEPC1-exosomes and (B) top 36 abundant protein in eEPC2-exosomes.

A. Top 27 abundant protein in eEPC1-exosomes

| mapped gene | annotation |
|---|---|
| CAT | Catalase; Occurs in almost all aerobically respiring organisms and serves to protect cells from th... |
| PCDHB4 | Protocadherin beta-4; Potential calcium-dependent cell-adhesion protein. May be involved in the ... |
| EEF1A1 | Elongation factor 1-alpha 1; This protein promotes the GTP-dependent binding of aminoacyl- tRN... |
| RARRES1 | Retinoic acid receptor responder protein 1; Inhibitor of the cytoplasmic carboxypeptidase AGBL2, ... |
| EEF2 | Elongation factor 2; Catalyzes the GTP-dependent ribosomal translocation step during translation... |
| PKP1 | Plakophilin-1; Seems to play a role in junctional plaques. Contributes to epidermal morphogenesis. |
| KRT73 | Keratin, type II cytoskeletal 73; Has a role in hair formation. Specific component of keratin interm... |
| CASP14 | Caspase-14 subunit p20, intermediate form; Non-apoptotic caspase involved in epidermal differe... |
| PKM | Pyruvate kinase PKM; Glycolytic enzyme that catalyzes the transfer of a phosphoryl group from p... |
| PRSS1 | Alpha-trypsin chain 1; Has activity against the synthetic substrates Boc-Phe-Ser- Arg-Mec, Boc-Le... |
| KRT78 | Keratin, type II cytoskeletal 78; Keratin 78; Belongs to the intermediate filament family. |
| CDSN | Corneodesmosin; Important for the epidermal barrier integrity. |
| FABP5 | Fatty acid-binding protein 5; Intracellular carrier for long-chain fatty acids and related active lipids... |
| DSP | Desmoplakin; Major high molecular weight protein of desmosomes. Involved in the organization ... |
| S100A9 | Protein S100-A9; S100A9 is a calcium- and zinc-binding protein which plays a prominent role in th... |
| CLIC4 | Chloride intracellular channel protein 4; Can insert into membranes and form poorly selective ion ... |
| DSG1 | Desmoglein-1; Component of intercellular desmosome junctions. Involved in the interaction of pl... |
| DSC1 | Desmocollin-1; Component of intercellular desmosome junctions. Involved in the interaction of pl... |
| CALML3 | Calmodulin-like protein 3; May function as a specific light chain of unconventional myosin-10 (MY... |
| TXN | Thioredoxin; Participates in various redox reactions through the reversible oxidation of its active ... |
| VIM | Vimentin; Vimentins are class-III intermediate filaments found in various non-epithelial cells, espe... |
| KRT19 | Keratin, type I cytoskeletal 19; Involved in the organization of myofibers. Together with KRT8, help... |
| KRT6B | Keratin, type II cytoskeletal 6B; Keratin 6B; Belongs to the intermediate filament family. |
| CSTA | Cystatin-A, N-terminally processed; This is an intracellular thiol proteinase inhibitor. Has an impor... |
| KRT6A | Keratin, type II cytoskeletal 6A; Epidermis-specific type I keratin involved in wound healing. Involv... |
| SERPINE1 | Plasminogen activator inhibitor 1; Serine protease inhibitor. Inhibits TMPRSS7. Is a primary inhibit... |
| IGFBP2 | Insulin-like growth factor-binding protein 2; Inhibits IGF-mediated growth and developmental rate... |

B. Top 36 abundant protein in eEPC2-exosomes

| mapped gene | annotation |
|---|---|
| PPIA | Peptidyl-prolyl cis-trans isomerase A, N-terminally processed; PPIases accelerate the folding of p... |
| ANXA5 | Annexin A5; This protein is an anticoagulant protein that acts as an indirect inhibitor of the throm... |
| HLA-B | HLA class I histocompatibility antigen, B alpha chain; Antigen-presenting major histocompatibilit... |
| FAP | Antiplasmin-cleaving enzyme FAP, soluble form; Cell surface glycoprotein serine protease that pa... |
| ITGAV | Integrin alpha-V heavy chain; The alpha-V (ITGAV) integrins are receptors for vitronectin, cytotacti... |
| CD9 | CD9 antigen; Integral membrane protein associated with integrins, which regulates different proc... |
| GNB1 | Guanine nucleotide-binding protein G(I)/G(S)/G(T) subunit beta-1; Guanine nucleotide-binding pro... |
| ITGB1 | Integrin beta-1; Integrins alpha-1/beta-1, alpha-2/beta-1, alpha-10/beta-1 and alpha-11/beta-1 are... |
| MME | Neprilysin; Thermolysin-like specificity, but is almost confined on acting on polypeptides of up to ... |
| VAMP3 | Vesicle-associated membrane protein 3; SNARE involved in vesicular transport from the late end... |
| GNA12 | Guanine nucleotide-binding protein subunit alpha-12; Guanine nucleotide-binding proteins (G prot... |
| ATP1A1 | Sodium/potassium-transporting ATPase subunit alpha-1; This is the catalytic component of the a... |
| BASP1 | Brain abundant membrane attached signal protein 1; Belongs to the BASP1 family. |
| DPP4 | Dipeptidyl peptidase 4 membrane form; Cell surface glycoprotein receptor involved in the costim... |
| YWHAQ | 14-3-3 protein theta; Adapter protein implicated in the regulation of a large spectrum of both gen... |
| STOM | Erythrocyte band 7 integral membrane protein; Regulates ion channel activity and transmembran... |
| LAMP2 | Lysosome-associated membrane glycoprotein 2; Plays an important role in chaperone-mediated ... |
| CLIC1 | Chloride intracellular channel protein 1; Can insert into membranes and form chloride ion channel... |
| BRIX1 | Ribosome biogenesis protein BRX1 homolog; Required for biogenesis of the 60S ribosomal subu... |
| ARF3 | ADP-ribosylation factor 3; GTP-binding protein that functions as an allosteric activator of the chol... |
| HLA-A | HLA class I histocompatibility antigen, A alpha chain; Antigen-presenting major histocompatibilit... |
| TMPRSS11A | Transmembrane protease serine 11A; Probable serine protease which may play a role in cellular s... |
| RAP1B | Ras-related protein Rap-1b; GTP-binding protein that possesses intrinsic GTPase activity. Contrib... |
| RRAS | Ras-related protein R-Ras; Regulates the organization of the actin cytoskeleton. With OSPBL3, mo... |
| CD63 | CD63 antigen; Functions as cell surface receptor for TIMP1 and plays a role in the activation of c... |
| BSG | Basigin; Plays an important role in targeting the monocarboxylate transporters SLC16A1, SLC16A... |
| SLC16A3 | Monocarboxylate transporter 4; Proton-linked monocarboxylate transporter. Catalyzes the rapid tr... |
| H2AC20 | Histone H2A type 2-C; Core component of nucleosome. Nucleosomes wrap and compact DNA int... |
| INTS12 | Integrator complex subunit 12; Component of the Integrator complex, a complex involved in the s... |
| CD151 | CD151 antigen; Essential for the proper assembly of the glomerular and tubular basement memb... |
| PFN1 | Profilin-1; Binds to actin and affects the structure of the cytoskeleton. At high concentrations, pro... |
| GJA1 | Gap junction alpha-1 protein; Gap junction protein that acts as a regulator of bladder capacity. A ... |
| ICAM1 | Intercellular adhesion molecule 1; ICAM proteins are ligands for the leukocyte adhesion protein L... |
| NPTN | Neuroplastin; Probable homophilic and heterophilic cell adhesion molecule involved in long term ... |
| VAT1 | Synaptic vesicle membrane protein VAT-1 homolog; Possesses ATPase activity (By similarity). Pl... |
| EHD1 | EH domain-containing protein 1; ATP- and membrane-binding protein that controls membrane reo... |
